# Integration of GWAS and transcriptome analyses to identify SNPs and candidate genes for aluminum tolerance in rapeseed (*Brassica napus* L.)

**DOI:** 10.1186/s12870-022-03508-w

**Published:** 2022-03-21

**Authors:** Huiwen Zhou, Xiaojun Xiao, Ali Asjad, Depeng Han, Wei Zheng, Guobin Xiao, Yingjin Huang, Qinghong Zhou

**Affiliations:** 1 Key Laboratory of Crop Physiology, Ecology and Genetic Breeding (Jiangxi Agricultural University), Ministry of Education/Jiangxi Province, Nanchang, 330045 Jiangxi Province China; 2 Institute of Jiangxi Oil-tea Camellia, Jiujiang University, Jiujiang, 332005 Jiangxi Province China; 3 Jiangxi Institute of Red Soil, Jinxian, 331717 Jiangxi Province China; 4 Department of Agriculture and Fisheries, PO Box 1054, Mareeba, QLD 4880 Australia

**Keywords:** *Brassica napus*, Aluminum, Genome-wide association study, Transcriptomic analysis, Candidate gene

## Abstract

**Background:**

The exchangeable aluminum (Al), released from the acid soils, is another addition to the environmental stress factors in the form of Al toxicity stress. Al stress affects the normal crop development and reduces the overall yield of rapeseed (*Brassica napus* L.). The response mechanism of plants to Al toxicity is complicated and difficult to understand with few QTL related studies in rapeseed under Al toxicity stress.

**Result:**

Using 200,510 SNPs developed by SLAF-seq (specific-locus amplified fragment sequencing) technology, we carried out the genome-wide association analysis (GWAS) in a population of 254 inbred lines of *B. napus* with large genetic variation and Al-tolerance differences. There were 43 SNPs significantly associated with eight Al-tolerance traits in the seedling stage were detected on 14 chromosomes, and 777 candidate genes were screened at the flanking 100 kb region of these SNPs. Moreover, RNA-seq detected 8291 and 5341 DEGs (the differentially expressed gene) in the Al -tolerant line (ATL) and -sensitive line (ASL), respectively. Based on integration of GWAS and RNA-seq analysis, 64 candidate genes from GWAS analysis differentially expressed at least once in 6 h vs 0 h or 24 h vs 0 h conditions in ATL or ASL. Moreover, four out of sixty-four candidate genes (*BnaA03g30320D*, *BnaA10g11500D*, *BnaC03g38360D* and *BnaC06g30030D*) were differentially expressed in both 6 h and 24 h compared to 0 h (control) conditions in both lines. The proposed model based on the candidate genes excavated in this study highlighted that Al stress disturb the oxidation-redox balance, causing abnormal synthesis and repair of cell wall and ABA signal transduction, ultimately resulting in inhibition of root elongation.

**Conclusions:**

The integration of GWAS and transcriptome analysis provide an effective strategy to explore the SNPs and candidate genes, which has a potential to develop molecular markers for breeding Al tolerant rapeseed varieties along with theoretical basis of molecular mechanisms for Al toxicity response of *Brassica napus* plants.

**Supplementary Information:**

The online version contains supplementary material available at 10.1186/s12870-022-03508-w.

## Introduction

Aluminum (Al), after oxygen and silicon, is the most abundant metal element in the earth’s crust. Al exists in the form of insoluble silicates or oxides which are less harmful to the growth and development of crops [1, 2]. However, soil pH value below 5.5 promotes exponential release of the exchangeable Al (mainly A1^3+^, Al(OH)^2+^ and Al(OH)_2_^+^) from silicates or oxides, which has a strong toxic effect on crop roots growth [3–5]. The free Al^3+^ ions can bind to the plasma membrane and nucleus, inhibiting the elongation and division of tip cells of crop roots, which affects the uptake of water and nutrients [6–8]. At present, about 40% of the world’s potentially arable lands are acidic (pH < 5.5) [9, 10]. Al toxicity has become a prominent factor affecting the crop growth on these acidic soils.

*Brassica napus* is the second largest oilseed crop in the world, providing edible oil for humans along with other multiple usages in the form of vegetable, forage, ornamental flower, honey and fertilizer [11, 12]. In China, the Yangtze River region where was the high rapeseed producing area has major issues with acidic soils specifically Al toxicity stress limiting the growth and seed yield of *B. napus* [4, 13]. At present, Al tolerance related research mainly focus on model plants such as *Arabidopsis*, rice, wheat and barley [14–16]. Previous research has demonstrated Al tolerance in plants as a complex trait controlled by multiple genes and pathways [17, 18]. Various genetic loci and genes involved in Al exclusion and tolerance mechanisms have been identified in model plants [18]. Success linked to a few genes (*BnALMT1*, *BnALMT2*, *CS* and *WMnSOD1*) in improving Al tolerance in transgenic plants has been studied previously [19–21]. However, many crucial genes linked to Al resistance in other crops, such as *RAL1* [22], *OsFRDL2* [23], *FeSTAR2* [24] and *VuSTOP1* [25], have not been reported in *B. napus.* It limits our understanding about the genetic variation and molecular mechanism of Al tolerance in *B. napus*.

Genome-wide association study (GWAS) have proved as a powerful tool in identification of desired trait linked genes in plants [26–28], and been applied in mining gene loci and candidate genes related to Al tolerance in various crops such as rice, wheat, and barley [14–16]. Recently, for *B. napus*, Gao et al. [13] detected 13 SNPs associated with two traits of relative root length and relative dry weight during germination using GWAS analysis. In addition, some studies have reported more accuracy and efficacy in screening the candidate genes for agronomic and stress-related traits by integrating GWAS and RNA-seq [29–32]. Zhang et al. [31] identified 16 loci significantly associated with water stress response in Canola using GWAS, and then 79 candidate genes were identified by combining differentially expressed genes (DEGs) detected by RNA-seq with loci from GWAS. Later on same approach identified 24 stalk rot resistance-related candidate genes in 17 sites, and 33 functional candidate genes related to rapeseed harvest index [30, 32]. Therefore, a combined strategy of GWAS and RNA-seq analysis showed more reliable potential to identify the candidate genes related to complex traits of rapeseed.

In this study, a set of 254 inbred lines of *B. napus* with large genetic variation and Al-tolerance differences were selected. This study also had an advantage of the 200,510 high-quality SNPs developed by SLAF-seq (specific-locus amplified fragment sequencing) technology [33]. GWAS was carried out to detect SNPs linked to the Al-tolerance and loci of elite allelic variation. In addition, the roots of two highly tolerant and susceptible rapeseed lines treated with Al ion stress were used to identify DEGs related to Al tolerance using RNA sequencing. DEGs for Al tolerance within the LD intervals containing significant SNP markers were selected as Al tolerance candidate genes by combining the analysis of GWAS and RNA-seq. The objective of this study was to identify SNP markers and candidate genes linked with Al tolerance in rapeseed.

## Results

### Phenotypic data

Eight traits for each of 254 rapeseed inbred lines were investigated under the stress of Al toxicity, and descriptive statistical analysis was summarized in Table S1 for fresh weight above ground, root average diameter, root fresh weight, root elongation, total root surface area, total root tip, total root volume, total root length of 254 rapeseed under CK and Al stress (Treatment). The coefficients of variation (CV, %) in CK and Treatment were ranged from 17.3 to 105.3 and 17.2 to 117.8, respectively.

**Fig. 1:**
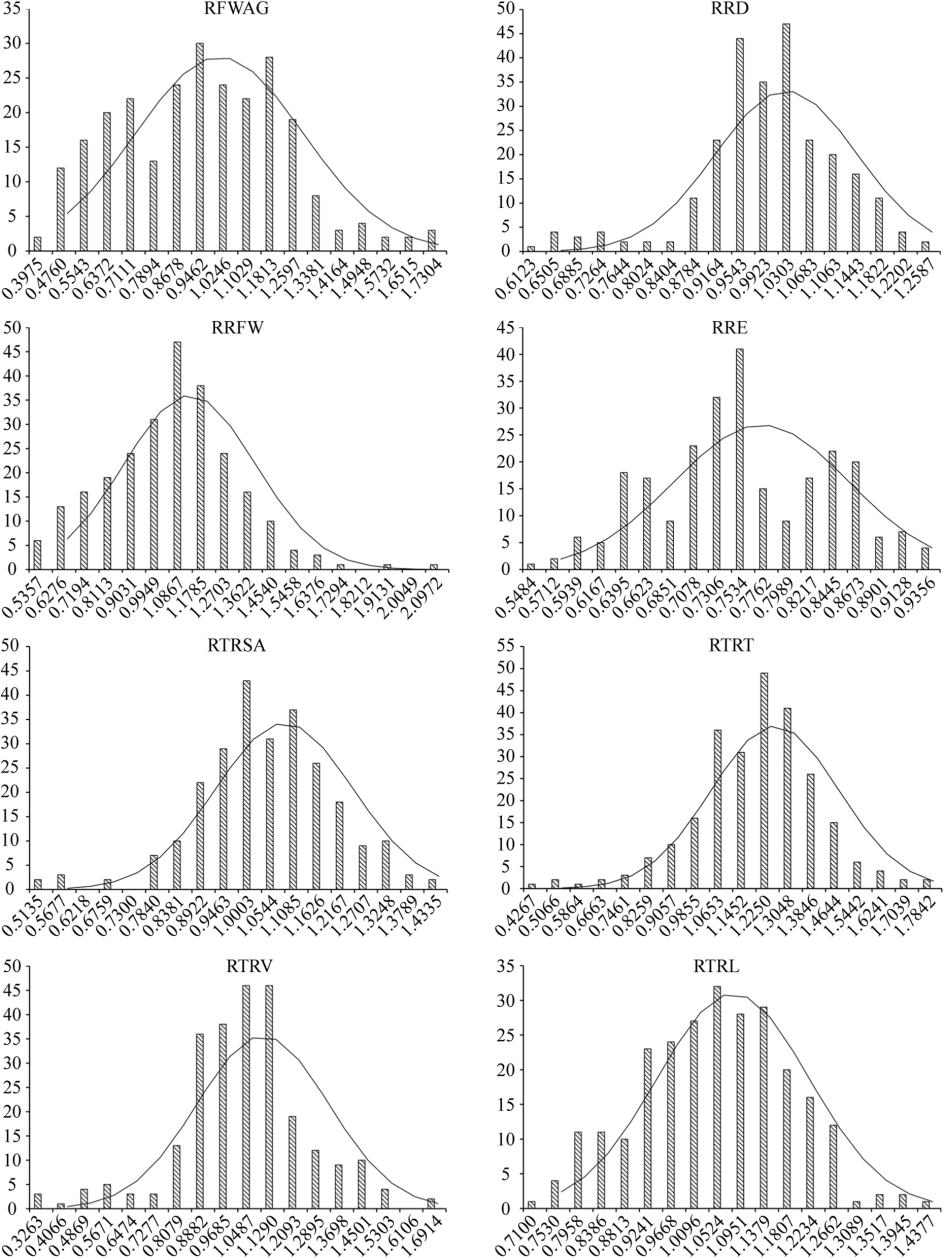
Frequency distribution of eight phenotypic traits related to Al tolerance in *Brassica napus*

The CV of eight traits between CK and Treatment ranged from 11.3 for relative root elongation (RRE) to 30.8 for relative root fresh weight (RRFW) (Table 1). RRE varied from 0.537 to 0.947 with an average of 0.750, and RRFW varied from 0.490 to 2.143 with an average of 1.071. The genotypes showed extremely significant differences with normal distribution for all the phenotypic traits among 254 inbred lines (Table 1; Fig. 1). This pattern showed presence of a broad phenotypic mutation under Al stress, which could effectively be used to locate Al tolerance linked candidate genes by GWAS.

**Table 1:** Statistical analysis of phenotypic traits in *Brassica napus*

Trait	Mean ± SD	Min	50% quantile	Max	CV/%	ANOVA		
						Repetition	Genotype	Error
RFWAG	0.920 ± 0.224	0.358	0.953	1.769	29.5	0.014	79.643^b^	1.940
RRD	1.008 ± 0.103	0.594	1.075	1.277	11.4	0.004	13.249^b^	4.945
RRFW	1.043 ± 0.209	0.490	1.071	2.143	30.8	0.726	111.458^b^	147.108
RRE	0.757 ± 0.085	0.537	0.750	0.947	11.3	0.004	7.3384^b^	1.0939
RTRSA	1.100 ± 0.109	0.487	1.042	1.460	15.1	0.039	25.125^b^	3.840
RTRT	1.215 ± 0.175	0.387	1.217	1.824	17.9	0.018	46.767^b^	6.262
RTRV	1.085 ± 0.181	0.286	1.049	1.731	21.5	0.119	51.130^b^	5.453
RTRL	1.082 ± 0.122	0.689	1.050	1.459	13.2	0.034	19.341^b^	5.273

Note: ^a^ and ^b^ mean significant difference at 0.05 and 0.01 levels respectively. RFWAG: relative fresh weight above ground; RRD: relative root average diameter; RRFW: relative root fresh weight; RRE: relative root elongation; RTRSA: relative total root surface area; RTRT: relative total root tip; RTRV: relative total root volume; RTRL: relative total root length

Furthermore, Strong positive correlations (*R* = 0.5–0.9) were observed among most of the traits, such as relative fresh weight above ground (RFWAG) with RRFW and relative total root tip (RTRT), relative total root surface area (RTRSA) with relative total root volume (RTRV), relative total root length (RTRL) and relative root average diameter (RRD) (Table 2).

**Table 2:** The correlation in eight traits studied under Al toxicity stress

	RFWAG	RRD	RRFW	RRE	RTRSA	RTRT	RTRV	RTRL
RFWAG	1							
RRD	−0.109	1						
RRFW	0.635^b^	0.058	1					
RRE	0.215^b^	−0.138^a^	0.140^a^	1				
RTRSA	0.487^b^	0.504^b^	0.413^b^	0.147^a^	1			
RTRT	0.294^b^	−0.411^b^	0.179^b^	0.199^b^	0.040	1		
RTRV	0.266^b^	0.809^b^	0.292^b^	0.017	0.865^b^	−0.157^a^	1	
RTRL	0.673^b^	−0.205^b^	0.492^b^	0.261^b^	0.669^b^	0.384^b^	0.329^b^	1

Note: ^a^ and ^b^ mean significant difference at 0.05 and 0.01 levels respectively

### Genome-wide association study

**Fig. 2:**
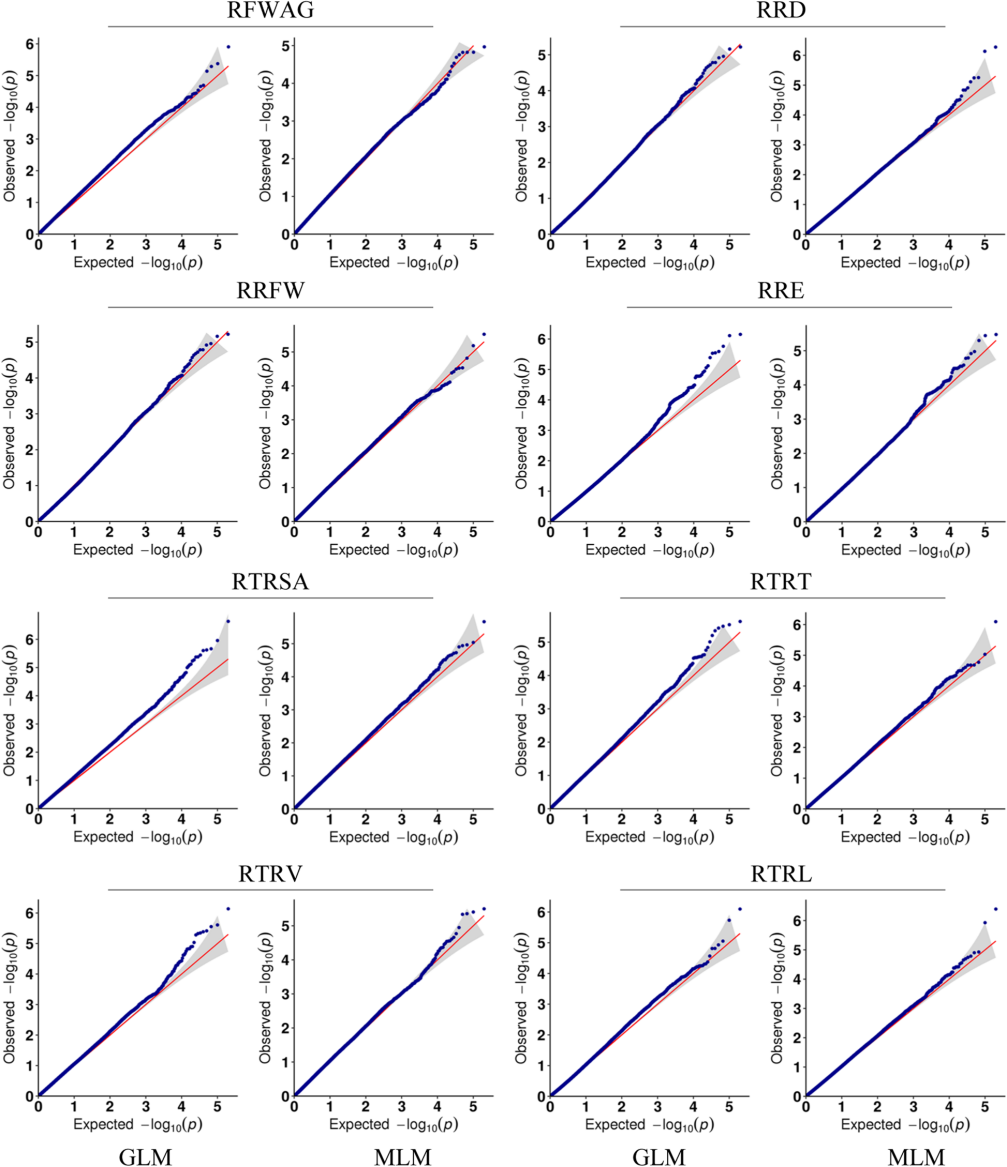
Quantile-quantile plots of estimated-lg (P) from association analysis of eight traits using GLM and MLM model

**Fig. 3:**
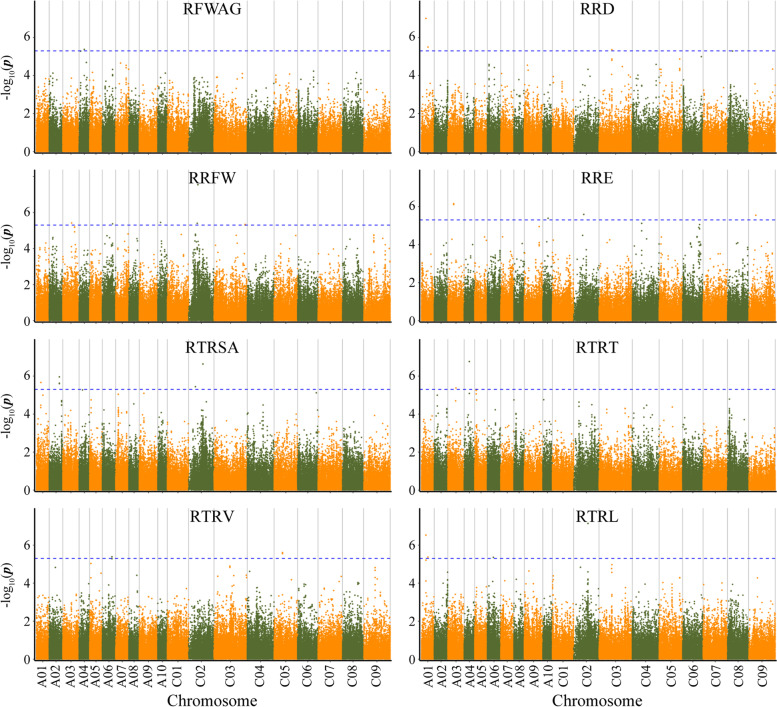
Manhattan plots for eight phenotypic traits related to Al tolerance in *Brassica napus* by GLM model. Note: The blue horizontal line represents that the extreme significance threshold -log10(P) value is approximately equal to 5.3

**Fig. 4:**
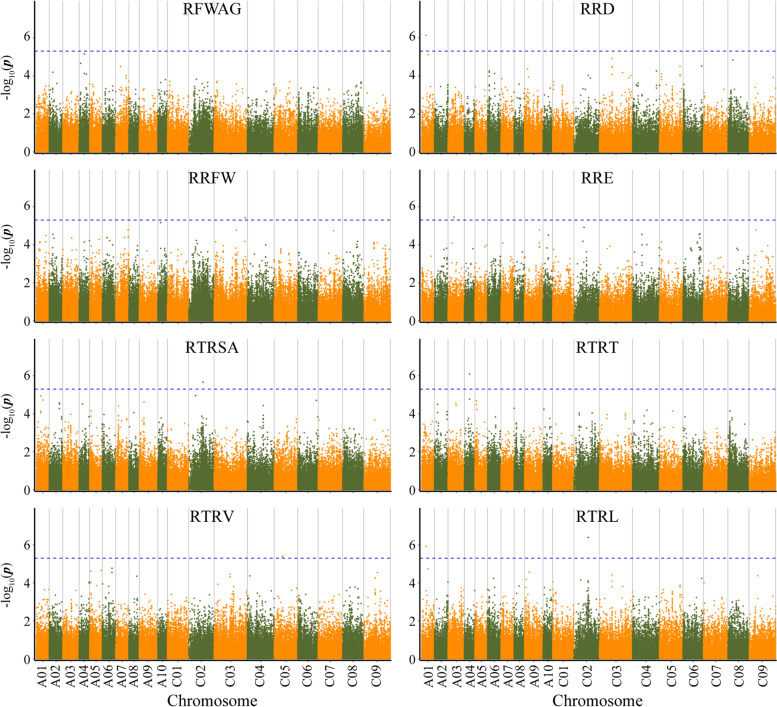
Manhattan plots for eight phenotypic traits related to Al tolerance in *Brassica napus* by MLM model. Note: The blue horizontal line represents that the extreme significance threshold -log10(P) value is approximately equal to 5.3

A total of 200,510 SNPs were used in the GWAS analysis for Al tolerance using GLM and MLM. The distribution agrees of *p*-values have a high consistency with observations by the Quantile-quantile plots (Q-Q plots) analysis (Fig. 2). The GLM analysis detected a total of 43 SNPs significantly associated with eight Al tolerance traits. These SNPs explained the phenotypic variation from 8.22 to 14.36% and their distribution was detected on 14 of the 19 *B. napus* chromosomes (excluding A05, A07, C01, C07 and C08). Besides, the largest number of significant SNPs was on chromosome C02 (six SNPs) and RRFW trait had the most associated SNPs (eight SNPs) (Fig. 3; Table 3). MLM analysis detected a total of 12 significantly associated SNPs with RRD (1), RRFW (3), RRE (2), RTRSA (1), RTRT (1), RTRV (2), and RTRL (1) on six chromosomes (A01, A03, A04, C02, C03 and C05), respectively, explaining phenotypic variation of 9.49 to 14.52% (Fig. 4; Table 3). Totally, 43 significant SNPs associated with eight Al tolerance traits were identified by GLM and MLM analyses.

**Table 3:** SNP loci significantly associated with eight traits of *Brassica napus* under Al toxicity stress

Traits	SNP	Chromosome	Position	*P* value	*R*^*2*^/%	Allele	GLM	MLM
RFWAG	*Bn-A04-p2852490*	A04	2,852,490	2.07E-06	11.32	T/A	●	
	*Bn-A04-p9422509*	A04	9,422,509	3.86E-06	11.25	G/A	●	
RRD	*Bn-A01-p8185115*	A01	8,185,115	9.87E-08 ~ 7.34E-07	14.02	T/C	●	○
	*Bn-A01-p11875598*	A01	11,875,598	3.18E-06	10.26	C/T	●	
	*Bn-C03-p23403794*	C03	23,403,794	4.33E-06	9.25	G/A	●	
RRFW	*Bn-A03-p16212704*	A03	16,212,704	3.81E-06	11.07	T/A	●	
	*Bn-A06-p18721845*	A06	18,721,845	4.24E-06	10.07	C/T	●	
	*Bn-A08-p15773789*	A08	15,773,789	4.21E-06	9.61	T/C	●	
	*Bn-A10-p5162750*	A10	5,162,750	3.54E-06	11.83	G/A	●	
	*Bn-C02-p15705371*	C02	15,705,371	3.97E-06	10.59	C/T	●	
	*Bn-C02-p16911048*	C02	16,911,048	3.40E-07 ~ 2.87E-08	14.00 ~ 14.36	C/T	●	○
	*Bn-C03-p56785277*	C03	56,785,277	4.90E-06 ~ 3.86E-06	9.64 ~ 10.74	C/T	●	○
	*Bn-C03-p56785578*	C03	56,785,578	4.57E-06 ~ 3.62E-06	9.69 ~ 10.82	A/G	●	○
RRE	*Bn-A03-p10703126*	A03	10,703,126	7.74E-07 ~ 3.62E-06	10.51 ~ 10.63	G/C	●	○
	*Bn-A03-p10703167*	A03	10,703,167	7.03E-07 ~ 3.35E-06	10.57 ~ 10.70	T/C	●	○
	*Bn-A10-p9658437*	A10	9,658,437	4.09E-06	11.63	A/T	●	
	*Bn-C02-p18429273*	C02	18,429,273	2.57E-06	11.14	T/G	●	
	*Bn-C04-p17161440*	C04	17,161,440	4.72E-06	10.91	G/A	●	
	*Bn-C06-p30829548*	C06	30,829,548	3.77E-06	10.17	C/T	●	
	*Bn-C09-p13036538*	C09	13,036,538	2.84E-06	9.97	G/A	●	
RTRSA	*Bn-A01-p8185115*	A01	8,185,115	2.16E-06	12	T/C	●	
	*Bn-A02-p18627325*	A02	18,627,325	2.38E-06	11.42	A/C	●	
	*Bn-A02-p18627333*	A02	18,627,333	2.50E-06	11.64	A/T	●	
	*Bn-A02-p18627380*	A02	18,627,380	1.10E-06	12.1	C/T	●	
	*Bn-A09-p6718215*	A09	6,718,215	3.98E-06	8.22	C/T	●	
	*Bn-C02-p12430774*	C02	12,430,774	3.61E-06	9.73	T/C	●	
	*Bn-C02-p26059415*	C02	26,059,415	2.31E-07 ~ 2.16E-06	11.11 ~ 11.70	G/C	●	○
RTRT	*Bn-A03-p5766579*	A03	5,766,579	4.56E-06	11.34	T/C	●	
	*Bn-A03-p14798182*	A03	14,798,182	4.23E-06	10.92	A/C	●	
	*Bn-A04-p9422509*	A04	9,422,509	8.05E-07 ~ 1.72E-07	13.83 ~ 14.07	G/A	●	○
	*Bn-A08-p3750050*	A08	3,750,050	4.54E-06	10.46	T/G	●	
	*Bn-A08-p3750315*	A08	3,750,315	3.78E-06	10.67	A/G	●	
	*Bn-C03-p16673270*	C03	16,673,270	1.01E-06	12.68	C/A	●	
RTRV	*Bn-A06-p17634684*	A06	17,634,684	4.12E-06	9.23%	G/T	●	
	*Bn-A06-p17634738*	A06	17,634,738	4.92E-06	9.07%	A/T	●	
	*Bn-C04-p4409586*	C04	4,409,586	3.39E-06	9.15	G/A	●	
	*Bn-C05-p15402975*	C05	15,402,975	4.41E-06 ~ 2.46E-06	9.49 ~ 9.98	G/A	●	○
	*Bn-C05-p15403018*	C05	15,403,018	3.94E-06 ~ 2.79E-06	9.40 ~ 10.08	A/C	●	○
RTRL	*Bn-A01-p8185115*	A01	8,185,115	2.97E-07 ~ 1.18E-06	13.63 ~ 14.52	T/C	●	○
	*Bn-A01-p11875598*	A01	11,875,598	4.27E-06	10.21%	C/T	●	
	*Bn-A06-p10959923*	A06	10,959,923	4.35E-06	9.60%	C/T	●	
	*Bn-A09-p8460525*	A09	8,460,525	2.43E-06	9.95%	C/T	●	
	*Bn-C02-p26059415*	C02	26,059,415	7.20E-08 ~ 4.05E-07	12.33 ~ 12.49	G/C	●	○

Note: R^2^ is the percentage of phenotypic variance explained by the SNP. ● indicates the GLM model detecting the significantly associated-trait SNP locus. ○ indicates the MLM model detecting the significantly associated-trait SNP locus

Among these significant SNPs (Table 3), two SNPs for RFWAG, detected by GLM analyses, were located on chromosomes A04. For RRD, three SNPs were identified on two chromosomes (A01 and C03), one SNP detected by GLM and two SNPs by both GLM and MLM models. Similarly, eight SNPs for RRFW were detected on chromosome A03, A06, A08, A10, C02 and C03, five of these were detected by GLM and three by both GLM and MLM analyses. GLM analysis was able to identify seven SNPs associated with RRE on six chromosomes (A03, A10, C02, C04, C06 and C09). GLM predictions were also confirmed by MLC identification of two SNPs on chromosome A03 associated with RRE. Six SNPs for RTRSA were detected on four chromosomes (A01, A02, A09 and C02) by only GLM analysis, however, one SNP was a shared result of both GLM and MLM analyses. MLM analysis detected six SNPs for RTRT, one SNP of which was consistent with GLM analysis. Five SNPs associated with RTRV were identified on chromosome A06, C04 and C05 by GLM analysis, two of which were consistent with MLM analysis. Five SNPs on  four chromosomes (A01, A06, A09 and C02) linked to RTRL were detected by GLM, and two SNPs detected by both GLM and MLM models.

Furthermore, four SNP loci were linked to multiple traits such as locus *Bn-A01-p8185115* (chromosome A01) was associated with three traits including RRD, RTRSA and RTRL. Similarly, *Bn-A01-p11875598* was associated with RRD and RTRL, *Bn-A04-p9422509* associated with RFWAG and RTRT, *Bn-C02-p26059415* associated with RTRSA and RTRL (Table 3).

### Identification of candidate genes

The candidate genes nearby the genome-wide significant SNPs were identified by using 100 kb flanking sequences of 43 SNPs (significantly associated with Al-tolerance) and ‘Darmor v4.1’ as the reference genome. This study resulted in the identification of 777 candidate genes, most of which genes were involved in various functions such as amino acid transport and metabolism, defense mechanisms, inorganic ion transport and metabolism by COG annotation (Fig. S1). Based on the functional annotations, some genes were known to be related with Al tolerance, such as MATE family proteins, ABC transporter family protein, aquaporin, sulfate transporter family protein, metal tolerance protein, glutathione S-transferase, xyloglucan endotransglucosylase/hydrolase protein and antioxidant proteins. Some candidate genes, such as *BnaC04g06050D*, *BnaC04g06060D*, *BnaA03g43560D*, *BnaA03g30320D* and *BnaA07g29670D*, mainly participated in the pathway related to transport and metabolism of inorganic ions, the transport and discharge of organic acids (citric acid), and oxidative stress response (Table S2).

### Transcriptome sequencing analysis

The transcriptomes of two breeding lines under Al stress were analyzed. More than 10.15 billion clean reads from 18 libraries of two genotypes were generated and mapped to the reference genome. The alignment results showed that 669 million reads of the clean reads were successfully mapped to the reference genome (Table S3).

To determine genes correlated with Al stress response, DEGs for 0 h, 6 h and 24 h Al stress treatments for both lines were identified. In the Al-tolerant line, a total of 3053 genes showed up-regulation and 3644 genes showed down-regulation under 6 h treatment, whereas 655 genes showed up-regulation and 939 genes showed down-regulation under 24 h treatment compared to 0 h treatment (FDR ≤ 0.05 and Log2(FC) ≥ 1.0 or ≤ − 1.0). Among these DEGs, 270 genes were up-regulated and 508 genes down-regulated under both of the 6 h and 24 h Al stress durations. An up-regulation was observed for 33 genes under 6 h duration, but 24 h duration down-regulated the same genes compared to control. However, 10 genes were down-regulated under 6 h but up-regulated under 24 h compared to control (Fig. S2A).

In the Al-sensitive line, a total of 2248 and 2058 genes showed up-regulation and down-regulation, respectively, under 6 h compared to control. 220 genes showed up-regulation and 815 genes showed down-regulation under 24 h treatment duration compared to control. Among these DEGs, 99 genes were up-regulated and 490 genes down-regulated, both under 6 h and 24 h Al stress. Compared to control treatment, five genes were up-regulated under 6 h, but down-regulated under 24 h (Fig. S2B). We randomly selected six genes involved in the Al stress for expression validation by qRT-PCR, and the expression trends were similar with the RNA-seq data (Fig. S3).

### DEGs of Al-tolerant and Al-sensitive lines

**Fig. 5:**
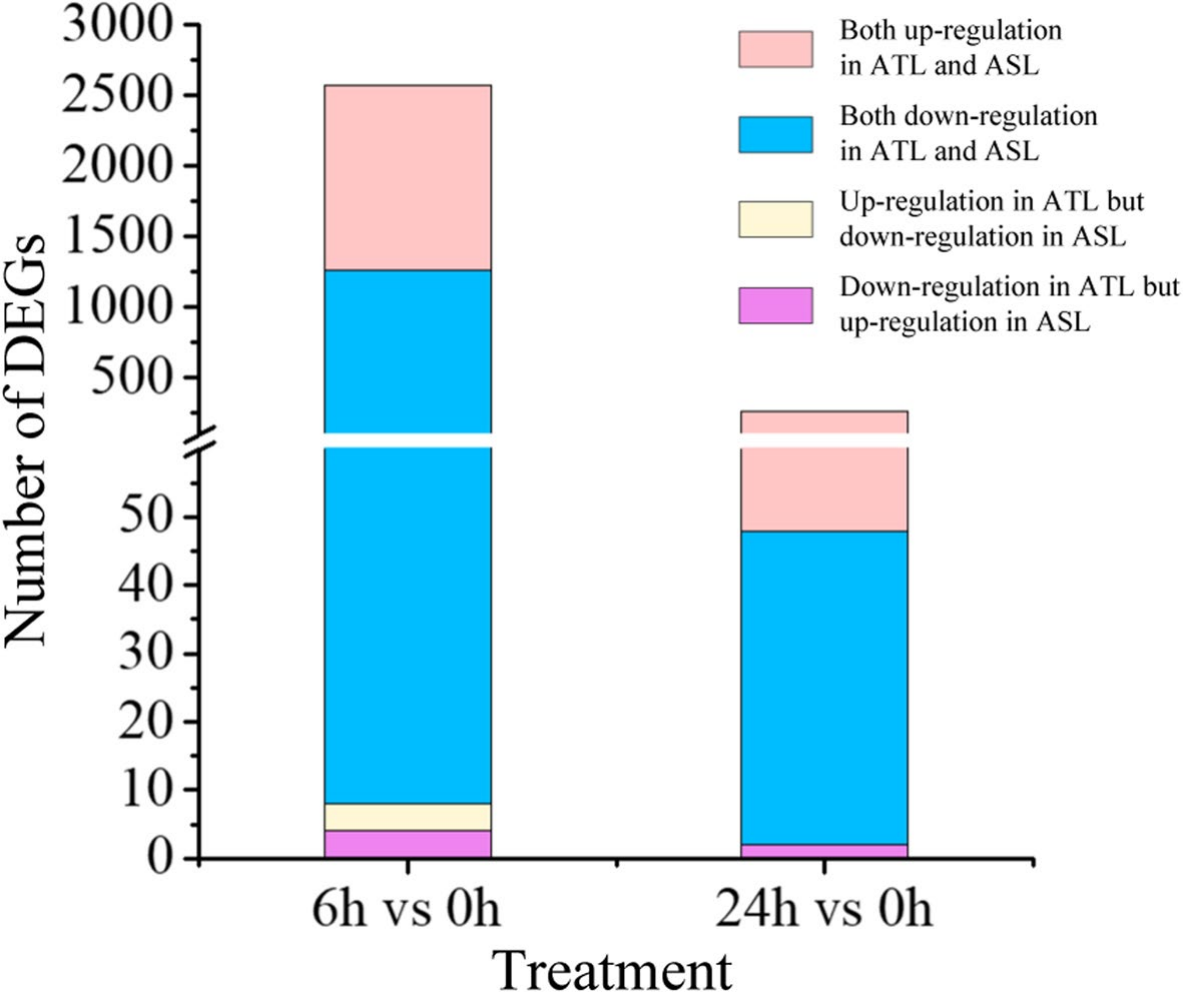
The number of up and down –regulation genes between ATL and ASL under Al toxicity

A total of 2569 DEGs under 6 h and 265 DEGs under 24 h treatment durations were compared to control for Al-tolerant and Al-sensitive lines, respectively. In 6 h treatment, 1255 genes were up-regulated and 1306 genes down-regulated both in ATL and ASL. Moreover, four genes were up-regulated in ATL and down-regulated in ASL; another group of four genes down-regulated in ATL but up-regulated in ASL in 6 h treatment (Fig. 5). The 24 h treatment duration compared to 0 h up-regulated 46 genes and down-regulated 217 genes in the ATL and ASL, two genes were up-regulated in ATL and down-regulated in ASL (Fig. 5). Interestingly, 151 common DEGs were identified in ATL and ASL under both treatment durations (Table S4). Among these DEGs, 25 DEGs and 124 DEGs were respectively up-regulated and down-regulated both in 6 h and 24 h of ATL and ASL, two DEGs were up-regulated in 6 h of ASL while down-regulated both in 6 h and 24 h of ATL and in 24 h of ASL.

Based on COG annotations, some genes of 151 DEGs were involved in carbohydrate transport and metabolism (20 DEGs), posttranslational modification (14 DEGs), Cell wall/membrane/envelope biogenesis (7 DEGs), signal transduction mechanisms (14 DEGs), defense mechanisms (13 DEGs), inorganic ion transport and metabolism (7 DEGs) (Fig. S4). Some of DEGs such as MATE family, ABC transporter family, zinc finger, glutathione S-transferase, xyloglucan endotransglucosylase/hydrolase protein and heavy-metal-associated domain were responsive to Al stress (Table S5).

### Identification of candidate genes by integrating GWAS and RNA-seq analysis

**Fig. 6:**
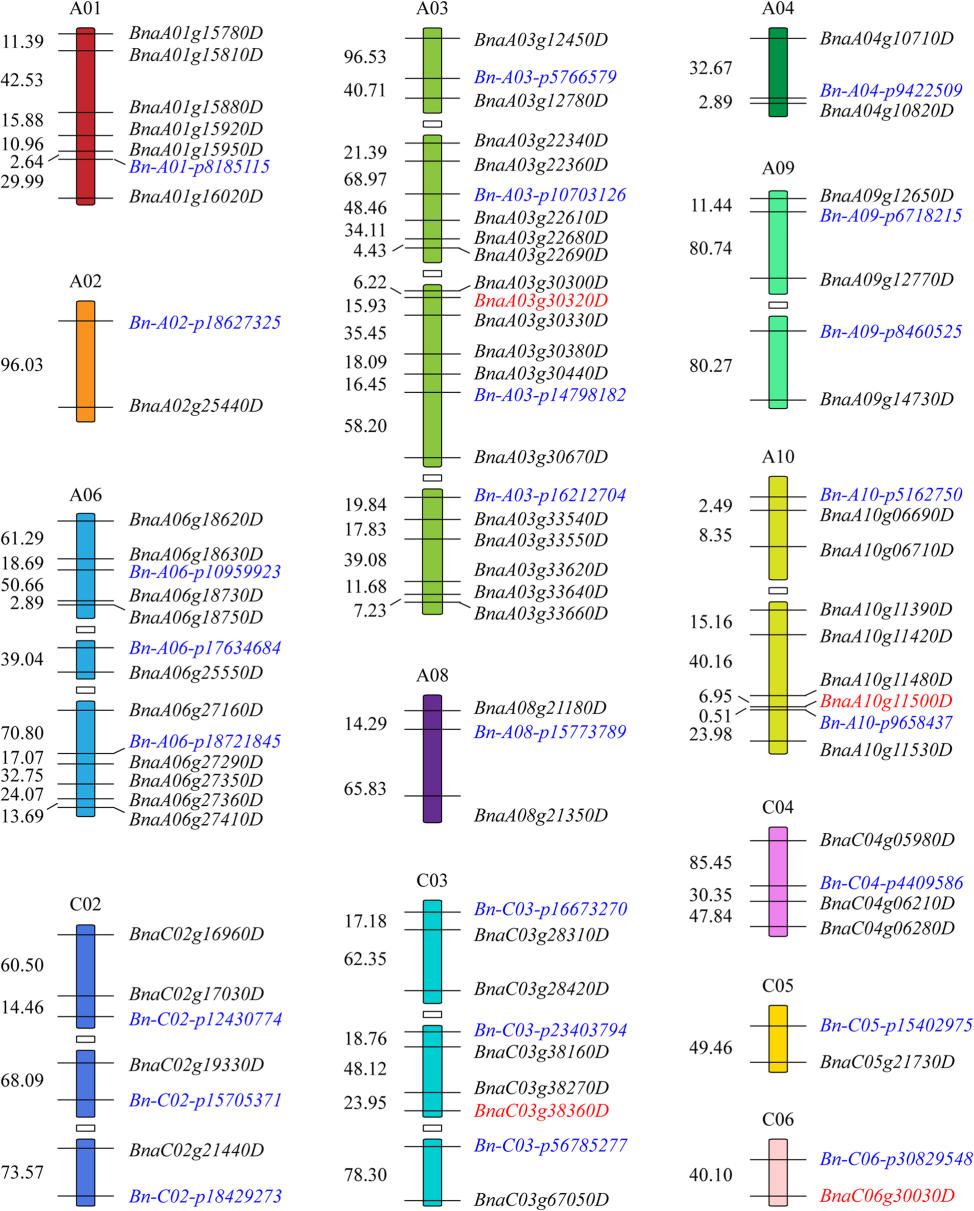
Distribution of candidate genes and their corresponding SNP loci associated with Al tolerance. Note: The blue as SNP loci, red as the gene differentially expressed both in 6 h vs 0 h and 24 h vs 0 h of ATL and ASL. The numeric values represent the relative distances in the genome, 1 = 1 kb

The potential candidate genes were prioritized by integrating DEGs obtained by GWAS and RNA-seq analysis. Out of 777 candidate genes identified by GWAS, 64 (8.24%) genes distributed on 13 of the 19 *B. napus* chromosomes (excluding A05, A07, C01, C07 C08 and C09). These candidate genes were differentially expressed in at least one genotype under 6 h or 24 h compared with 0 h conditions (Fig. 6; Table S6). The largest number of candidate genes were on chromosome A03 (18 genes) in the flanking 100 kb region of four SNPs. Ten candidate genes were screened from three SNPs and seven genes from two SNPs on chromosome A06 and A10, respectively. Similarly, six candidate genes were screened from the flanking region of one SNP (*Bn-A01-p8185115*) on chromosome A01.

**Fig. 7:**
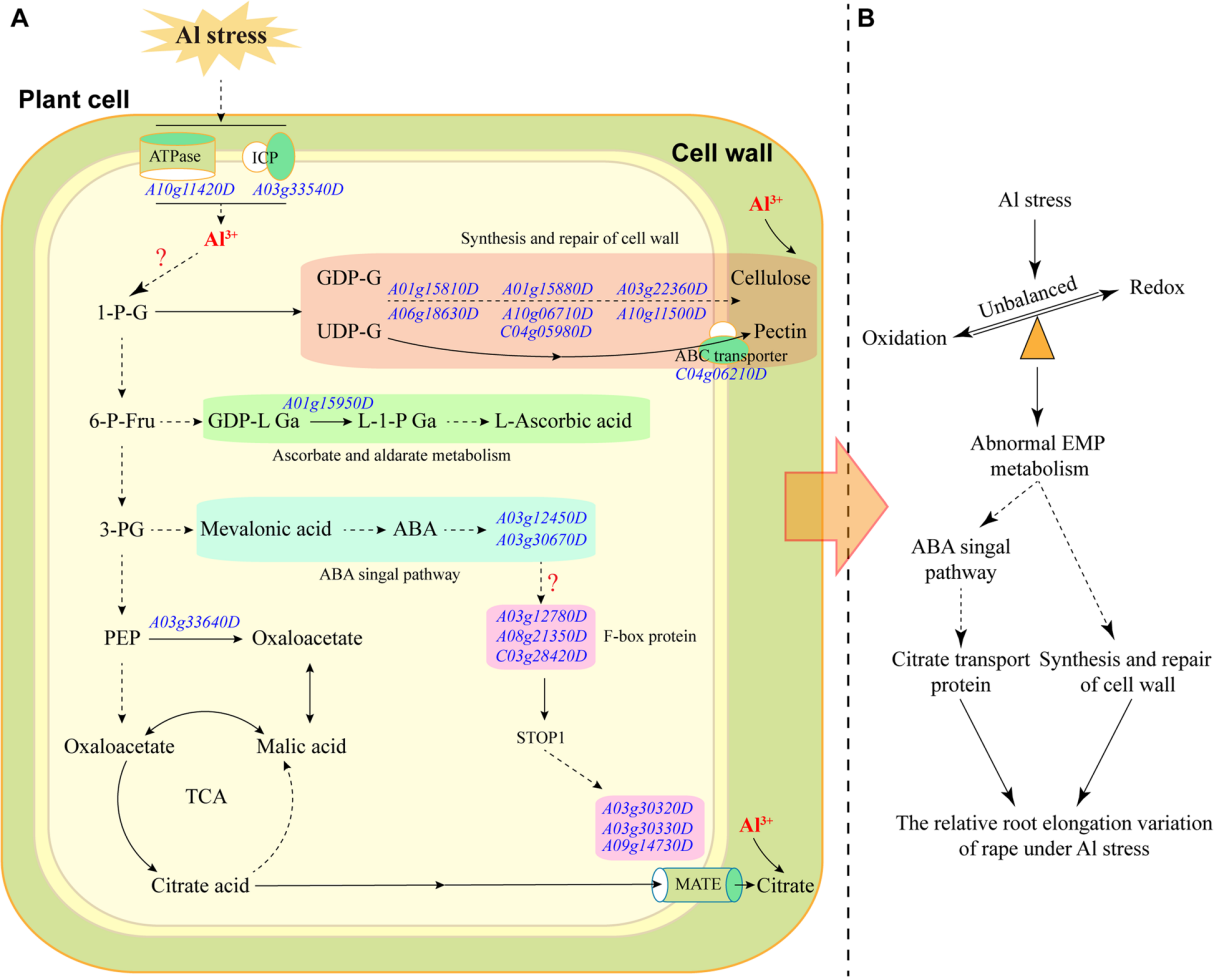
The possible metabolic pathway for candidate gens under Al stress (**A**) and the possible model for rapeseed in response to Al stress (**B**). Note: 1-P-G, glucose 1-phosphate; 6-P-Fru, fructose 6-phosphate; GDP-G, GDP-glucose; UDP-G, UDP-glucose; GDP-L Ga, galactose GDP-L; L-1-P Ga, galactose L-1-phosphate; 3-PG, 3-phosphoglycerate; PEP, phosphoenolpyruvate; ICP, ion channel protein; STOP1, sensitive to proton rhizotoxicity 1; MATE, MATE efflux family protein; ABA, abscisic acid; TCA, tricarboxylic acid cycle; EMP pathway, glycolysis pathway

The candidate genes enabled us to identify several Al tolerance related genes in breeding lines (Fig. 6; Fig. 7). For example, two genes (*BnaA03g30330D* and *BnaA03g30320D*) were detected adjacent to SNP *Bn-A03-p14798182* and another gene (*BnaA09g14730D*) adjacent to SNP *Bn-A09-p8460525* belong to the MATE gene family. A gene *BnaC04g06210D* on chromosome C04 belonged to ABC transporter family protein in the vicinity of SNP *Bn-C04-p4409586*. One gene of *BnaA03g12450D* involved in abscisic acid (ABA) signal regulation was found in the vicinity of SNP *Bn-A03-p5766579*. These genes *BnaA01g15810D, BnaA01g15880D, BnaA03g22360D, BnaA06g18630D, BnaA10g06710D, BnaA10g11500D, BnaC04g05980D* were located adjacent to SNP *Bn-A01-p8185115*, *Bn-A03-p10703126*, *Bn-A06-p10959923*, *Bn-A10-p5162750*, *Bn-A10-p9658437* and *Bn-C04-p4409586* on their respective chromosomes and participated in cell wall development. Besides, two genes (*BnaA03g22680D* and *BnaA03g33540D*) on chromosome A03 involved in ion transport process.

Among these 64 candidate genes, four were simultaneously detected by GWAS and the common DEGs between 6 h vs 0 h and 24 h vs 0 h in ATL and ASL. These four genes include those encoding a MATE family protein (*BnaA03g30320D*), xyloglucan endotransglucosylase/hydrolase (*BnaA10g11500D*), a DnaJ domain protein (*BnaC03g38360D*), and an unknown function protein (*BnaC06g30030D*) (Table 4).

**Table 4:** The gene differentially expressed both in 6 h vs 0 h and 24 h vs 0 h of ATL and ASL

Gene ID	Chr	SNP	Relative expression level				Function description
			6 h vs 0 h in ATL	24 h vs 0 h in ATL	6 h vs 0 h in ASL	24 h vs 0 h in ASL	
*BnaA03g30320D*	A03	*Bn-A03-p14798182*	−2.89	−1.03	−3.49	−2.22	MATE family protein
*BnaA10g11500D*	A10	*Bn-A10-p9658437*	−2.46	−2.83	−2.29	−2.96	xyloglucan endotransglucosylase/hydrolase
*BnaC03g38360D*	C03	*Bn-C03-p23403794*	2.22	1.06	1.98	1.14	DnaJ domain protein
*BnaC06g30030D*	C06	*Bn-C06-p30829548*	−2.38	−1.64	−1.98	−1.41	function unknown

## Discussion

### Detection of novel SNP loci significantly associated with Al tolerance in *B. napus*

Al toxicity stress limits the crop growth and yield by affecting root meristem, cell mitosis and root growth in crops [8, 34]. Al toxicity has been reported in various crops targeting root related traits such as longest and primary root growth, total root growth and relative root elongation for discovering genes involved in Al tolerance by GWAS approach [13, 35, 36]. For example, Famoso et al. [16] identified 48 regions associated with three root growth parameters in rice seedlings; later on this finding helped in identification of 23 and 43 significant loci associated with relative root elongation in rice [35, 36]. Previously, 13 SNPs significantly associated with relative root length and relative dry weight during germination period detected in 169 rapeseed cultivars (lines) using 60 K Brassica Illumina Infinium SNP array [13]. In this research, we investigated eight traits related to Al tolerance (RFWAG, RRD, RRFW, RRE, RTRSA, RTRT, RTRV and RTRL) for 254 rapeseed accessions and detected 43 associated SNP loci on 14 chromosomes by GWAS using SLAF-Seq as detailed in our previous studies [33, 37], which explained the phenotypic variation from 8.22 to 14.36% (Fig. 2; Table 3). Among these SNP loci, *Bn-A04-p9422509* and *Bn-A09-p8460525* were respectively in the range 1 Mb of *Bn-A04-p7776319* and *Bn-A09-p9030563* which were significantly associated with Al tolerance at germination stage of *B. napus* in previous study [13]. In addition, there were 41 novel SNP loci discovered on 12 chromosomes (Table 3), and four SNPs of which (*Bn-A01-p8185115*, *Bn-A01-p11875598*, *Bn-A04-p9422509* and *Bn-C02-p26059415*) were significantly associated with more than one trait, which might be caused by linkage or pleiotropy [38]. In previous studies, the RRE were used to evaluate the Al resistance [35, 39, 40], in this study, the RTRL were positive correlations with RRE, and five SNP loci were significantly associated with RTRL, three of five SNP loci were significantly associated with multiple traits. Therefore, RRE and RTRL can be used to evaluate the Al resistance of *B. napus*. Our results provide insights into the significantly association of SNPs with Al tolerance traits, which could be a potential marker for improving the Al tolerance breeding in *B. napus*.

### Mining of candidate genes to uncover the Al tolerance gene network by integrating GWAS and transcriptome

Two main strategies have been found to deal with Al toxicity in acidic soils, including external exclusion and internal tolerance [17, 18, 41]. The external exclusion prevents plant roots absorbing a large amount of Al (Al^3+^) to reduce toxicity [17, 42]. The internal tolerance mechanism detoxify internal Al in plant cells by chelating with organic acids and converting the absorbed ionic Al into combined Al [17, 43]. Among these strategies, secretion of Al-induced root organic acid to chelate Al for protecting cell wall from Al binding is the well-documented mechanism [17, 44, 45]. Previously, map-based cloning found MATE gene family to resist Al toxicity stress in barley (*HvAACT*) and sorghum (*SbMATE*) [46, 47]. It was followed by MATE homologs to promote citrate excretion into the rhizosphere to protect roots from Al toxicity in maize (*ZmMATE1*) [48], rice (*OsFRDL4* and *OsFRDL2*) [23, 49] and soybean (*GmMATE75*, *GmMATE79* and *GmMATE87*) [50]. In current study, five genes belonging to MATE gene family were detected by GWAS, three candidate genes (*BnaA03g30320D* and *BnaA03g30330D* adjacent to SNP *Bn-A03-p14798182*, *BnaA09g14730D* adjacent to SNP *Bn-A09-p8460525*) were differentially expressed by RNA-seq (Fig. 7A). The expression of the gene *BnaA03g30320D* was more down-regulated in ASL than ATL under both 6 h vs 0 h and 24 h vs 0 h conditions. Our results are consistent with a previous detection of MATE family genes at germination stage under Al toxicity stress [13], which contribute in Al tolerance in *B. napus*.

The root cell wall becomes the next site of Al interaction after traversing the organic acid barrier in the root rhizosphere [18]. Even with a complex structure of cell wall, the negatively charged carboxyl groups in pectin and uncharged hemicellulose binding, resulted in the distortion of cell wall extension under Al stress [51, 52]. In the present study, one ABC transporter gene on chromosome C04 (*BnaC04g06210D*) was identified, which is involved in abscisic acid (ABA) transport and responses [53]. ABC transporter could regulate the plant Al tolerance by transporting UDP-glucose, which affects hemicellulose metabolism by regulating xyloglucan endotransglucosylase/hydrolases activity [24, 54]. ABC transporters also play an important role in Al resistance mechanism [53, 55, 56], for instance, *OsALS1*, *FeALS1.1* and *FeALS1.2*, all of the homologous gene *AtALS1* encoding a half-size ABC transporter, were involved in the internal detoxification of Al in rice and buckwheat [57, 58]. Furthermore, seven candidate genes (*BnaA01g15810D* and *BnaA01g15880D* adjacent to *Bn-A01-p8185115*, *BnaA03g22360D* adjacent to *Bn-A03-p10703126*, *BnaA06g18630D* adjacent to *Bn-A06-p10955523*, *BnaA10g06710D* adjacent to *Bn-A10-p5162750*, *BnaA10g11500D* adjacent to *Bn-A10-p9658437*, and *BnaC04g05980D* adjacent to *Bn-C04-p4409586*) involved in cell wall components were also detected in this study (Fig. 7A). Two of seven genes encoding xyloglucan endotransglucosylase/hydrolase (*BnaA06g18630D* and *BnaA10g11500D*) were detected on chromosome A06 and A10, respectively. Previously, Zhu et al. [51] reported that *XTH31* encoding a xyloglucan endotransglucosylase/hydrolases regulates Al sensitivity by modulating cell wall xyloglucan content and Al binding capacity. Both ASL and ATL showed down-regulation of *BnaA10g11500D* in 6 h vs 0 h and 24 h vs 0 h conditions. ATL also showed a down-regulation of *BnaA10g06710D* (probable pectinesterase/pectinesterase inhibitor) in 6 h vs 0 h and 24 h vs 0 h conditions, indicating the presence of gene specific expression pattern.

Plant hormones such as ABA, JA and SA play an important role in the stress related defense system [59]. ABA signal transduction pathways provide an additional layer of regulatory control over Al tolerance in plants [60–62]. Furthermore, the exogenous application of ABA could increase the activity of citrate synthase and decrease Al accumulation [60, 61]. In this study, three candidate genes (*BnaA03g12450D* in the vicinity of *Bn-A03-p5766579*, *BnaA03g30670D* in the vicinity of *Bn-A03-p14798182*, and *BnaC04g06210D* in the vicinity of *Bn-C04-p4409586*) related to ABA signal pathway were identified on chromosome A03 and C04, respectively (Fig. 7A). One candidate gene, *BnaA03g12450D* encodes ABA receptor *PYL8* with up-regulation in 6 h vs 0 h condition in both ASL and ATL. In addition, various other defense related genes were detected including *BnaA03g12780D* in the vicinity of *Bn-A03-p5766579*, *BnaA08g21350D* in the vicinity of *Bn-A08-p15773789* and *BnaC03g28420D* in the vicinity of *Bn-C03-p16673270* which encode for F-box proteins, and *BnaA06g27360D* encodes zinc finger protein (Fig. 7A). A C2H2-type zinc finger protein *STOP1* as the major factor regulating *MATE1* expression plays a critical role in Al tolerance, and the F-box protein *RAE1* regulates the stability of the Al-resistance transcription factor *STOP1* [25, 63, 64]. Further research on these genes will reveal their roles under Al stress in *B. napus*.

## Conclusions

A total of 43 SNP loci significantly associated with 8 phenotypic traits related to Al toxicity stress were detected on 14 chromosomes of *B. napus* by GWAS. Further exploration of SNP flanking regions discovered 777 candidate genes. RNA-seq approach detected 8291 and 5341 DEGs in ATL and ASL, respectively. Integration of GWAS and RNA-seq results found 64 differentially expressed candidate genes under 6 h and/or 24 h compared to control conditions. Among candidate DEGs, *BnaA03g30320D* and *BnaA10g11500D* encode MATE family protein and xyloglucan endotransglucosylase/hydrolase, respectively, which are responsive to Al toxicity stress. In addition, the proposed model showed that the oxidation-redox balance was perturbed under Al stress, causing abnormal cell wall repair and ABA signal transduction, ultimately leading to inhibition of root elongation. These exploratory analyses of Al toxicity linked candidate genes by integrating GWAS and RNA-seq showed a great power in uncovering genetic variation in Al toxicity stress in rapeseed. This strategy would be useful in understanding the molecular mechanisms responding Al toxicity. Furthermore, knowledge on the level of Al tolerance in rapeseed along with the associated SNPs from this research, would be useful for breeding future Al tolerant varieties.

## Materials and methods

### Plant materials and growth conditions

In this study, 254 oilseed rapeseed inbred lines were collected and preserved in the Key Laboratory of Crop Physiology, Ecology and Genetic Breeding (Jiangxi Agricultural University), Ministry of Education/Jiangxi Province. Of these oilseed rapeseed inbred lines, 220 lines were Semi-winter types, 15 lines were Spring types and 19 lines were Winter types. In total, 237 lines were collected from China, seven lines from Europe, five lines from Japan and five lines from Canada. The pertinent information for all accessions is shown in Table S7. These lines were grown under controlled conditions using growth chambers with 14 h light at 25 °C/20 °C (day/night) temperature.

### Phenotyping for Al stress

Seeds of uniform size were selected from 254 accessions and separately surface sterilized in 1% hydrogen peroxide for 30 min [65]. Then, seeds were washed with ultrapure water for three times before spreading on the gauze cloth. In order to adapt the seedlings to total nutrient solution environment betterly, the uniform and healthy rapeseed seedlings before the lateral roots differentiation from the main roots were sequentially transferred into 1/4, 1/2 and total Hoagland’s nutrient solutions without Al treatment gradully. Each nutrient solution was cultured for 3 days. After transplanted into 0.5 mmol·L^− 1^ CaCl_2_ solution (pH 4.5) for 12 h, the seedlings were exposed to the nutrient solution (pH 4.5) containing 100 μmol·L^− 1^ AlCl_3_ for 28 days for Al stress treatment. The seedlings exposed to the total Hoagland’s nutrient solution (pH 4.5) with 0 μmol·L^− 1^ AlCl_3_ were used as a control. The pH of the solution was adjusted every 2 days and was kept 4.5 with NaOH or HCl. Each treatment had four biological replicates.

After 28 days, the fresh weight (g) of the above and below ground, and main root length (cm) were measured. Then, the root system was scanned by RhizoScan (Regent, Canada). The total root length (cm), root surface area (cm^2^), average root diameter (mm), root volume (cm^3^) and number of root tips of each material were analyzed by root image analysis software WinRHIZO STD4800 LA2400. The relative values (ratio) of each trait between treatments and controls were statistically analyzed using Excel and DPS, including relative fresh weight above ground (RFWAG), relative root average diameter (RRD), relative root fresh weight (RRFW), relative root elongation (RRE), relative total root surface area (RTRSA), relative total root tip (RTRT), relative total root volume (RTRV) and relative total root length (RTRL). The relative value was calculated by following formula: (the data of each phenotyping under Al treatment) / (the data of each phenotyping in control).

### GWAS analysis

Based on the 200,510 SNPs developed in our previous research [33, 37], GWAS analysis for eight traits was carried out using generalized linear models (GLM) and mixed linear models (MLM) in Tassel 5.0 software [66]. GLM was adjusted using the Q-matrix which was calculated by the Admixture software package [67], MLM using Q-matrix and K-matrix was predicted by the SPAGeDi software [68]. The Quantile-Quantile plot (Q-Q plot) and the Manhattan plot were drawn by the GGplot2 software [69] and QQman software [70], respectively. The threshold value of -log_10_(P), set as -log_10_(1/200,510 SNPs), is approximately equal to 5.3 for significantly correlating SNPs.

To screen the candidate genes related to the Al tolerance, significant SNPs which were closely linked to the eight traits were mapped to the reference rapeseed genome [71]. The 100 kb flanking regions on either side of these SNPs were used to identify candidate genes. All candidate genes were selected based on GO (http://geneontology.org/), COG (https://www.ncbi.nlm.nih.gov/research/cog-project/), NR (ftp://ftp.ncbi.nih.gov/blast/db/FASTA/), SwissProt (http://www.expasy.org/sprot/) and KEGG (https://www.genome.jp/kegg/) databases.

### RNA-seq under Al stress and data analysis

For RNA-seq, the Al -tolerant (ATL, FDH188) and -sensitive (ASL, FDH152) lines screened from 254 accessions in our previous research [72] were as the materials (Fig. S5). Two varieties were treated with 150 μmol·L^− 1^ AlCl_3_ for 0 h (control), 6 h and 24 h, respectively. Then the roots were quickly frozen in liquid nitrogen. Each treatment had three biological replicates.

To detect the DEGs, the low-quality reads with an ‘N’ percentage over 5% and more than 20% bases with a Q-value < 20 were removed by Perl program. The retained high quality reads were mapped to the reference rapeseed genome by Tophat [73], and then assembled by Cufflinks [74]. The genes expression levels were normalized by The Fragments Per Kb per Million fragments (FPKM) values. The genes with FPKM values ≤ 0.5 of all libraries were removed. False discovery rate (FDR) < 0.05 and log_2_(fold change (FC)) ≥ 1.0 or ≤ − 1.0 were used to determine the significantly DEGs. Combining the analysis of GWAS and RNA-seq, DEGs for Al tolerance within in the 100 kb intervals containing significant SNP markers will be selected as Al tolerance candidate genes.

The raw read data reported in this study have been deposited in the Genome Sequence Archive (GSA) in the National Genomics Data Center, under submission ID CRA003428 (https://ngdc.cncb.ac.cn/gsa/browse/CRA003428).

### Validation of RNA-seq by qRT-PCR

Total RNA of 18 samples under 150 μmol·L^-1^ AlCl_3_ for 0 h, 6 h and 24 h between ATL and ASL were extracted by MiniBEST Universal RNA Extraction Kit (TaKaRa) followed by construction of cDNA libraries using PrimeScript™ RT Master Mix (TaKaRa). Expression of six DEGs was determined with TB Green® Premix Ex Taq™ II (TaKaRa) by eppendorf realplex^2^ (Eppendorf, Germany). The primer sequences for qRT-PCR are listed in Table S8. The relative expression levels were calculated using the 2^-△△Ct^ method based on the normalization to the reference genes *ACT6*. Three technical replicates were performed for DEGs and reference genes.

## Supplementary Information

**Additional file 1.**

**Additional file 2.**

**Additional file 3.**

**Additional file 4.**

**Additional file 5.**

**Additional file 6.**

**Additional file 7.**

**Additional file 8.**

**Additional file 9.**

**Additional file 10.**

**Additional file 11.**

**Additional file 12.**

**Additional file 13.**

## Data Availability

The datasets of RNA-seq during the current study are available in the Genome Sequence Archive (GSA) in the National Genomics Data Center repository (submission ID CRA003428, https://ngdc.cncb.ac.cn/gsa/browse/CRA003428). The data sets supporting the results of this article are included within the article and its additional files. The rapeseed cultivars and breeding materials are included within the article and its additional files (Table S7).

## Abbreviations

GWAS: genome-wide association study
ATL: the Al-tolerant line
ASL: the Al-sensitive line
*B. napus*: *Brassica napus*
DEGs: the differentially expressed genes
SLAF-seq: specific-locus amplified fragment sequencing
RFWAG: relative fresh weight above ground
RRD: relative root average diameter
RRFW: relative root fresh weight
RRE: relative root elongation
RTRSA: relative total root surface area
RTRT: relative total root tip
RTRV: relative total root volume
RTRL: relative total root length
FPKM: The Fragments Per Kb per Million fragments
FDR: False discovery rate
FC: fold change
GSA: the Genome Sequence Archive
