# Supplementary figures and images for: Integration of GWAS and transcriptome analyses to identify SNPs and candidate genes for aluminum tolerance in rapeseed (Brassica napus L.)
Source: BMC Plant Biol. 2022 Mar 21;22:130. doi: 10.1186/s12870-022-03508-w (PMC8935790; doi:10.1186/s12870-022-03508-w)

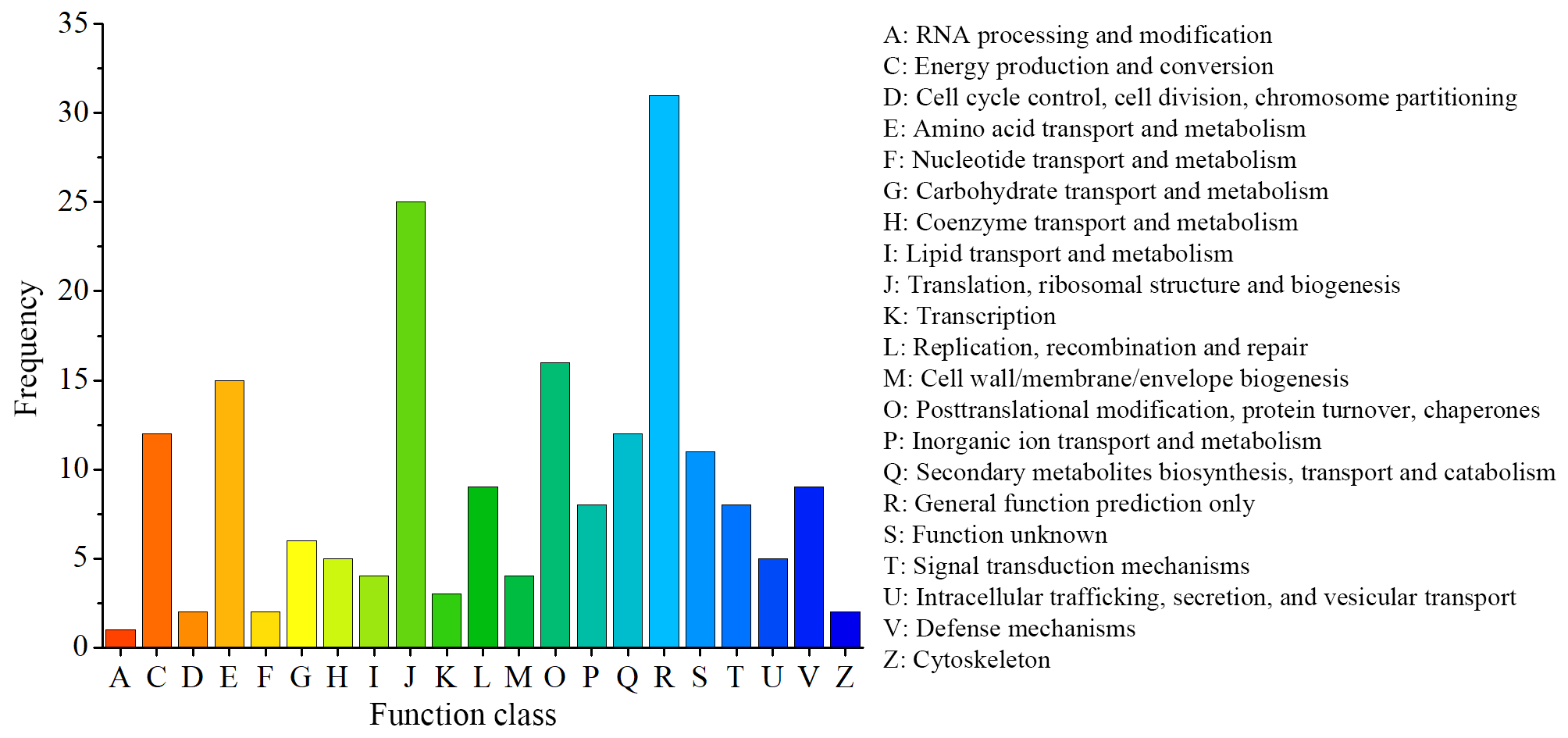

Supplement: Supplementary file 1 — Additional file 1. [file 12870_2022_3508_MOESM1_ESM.tif]

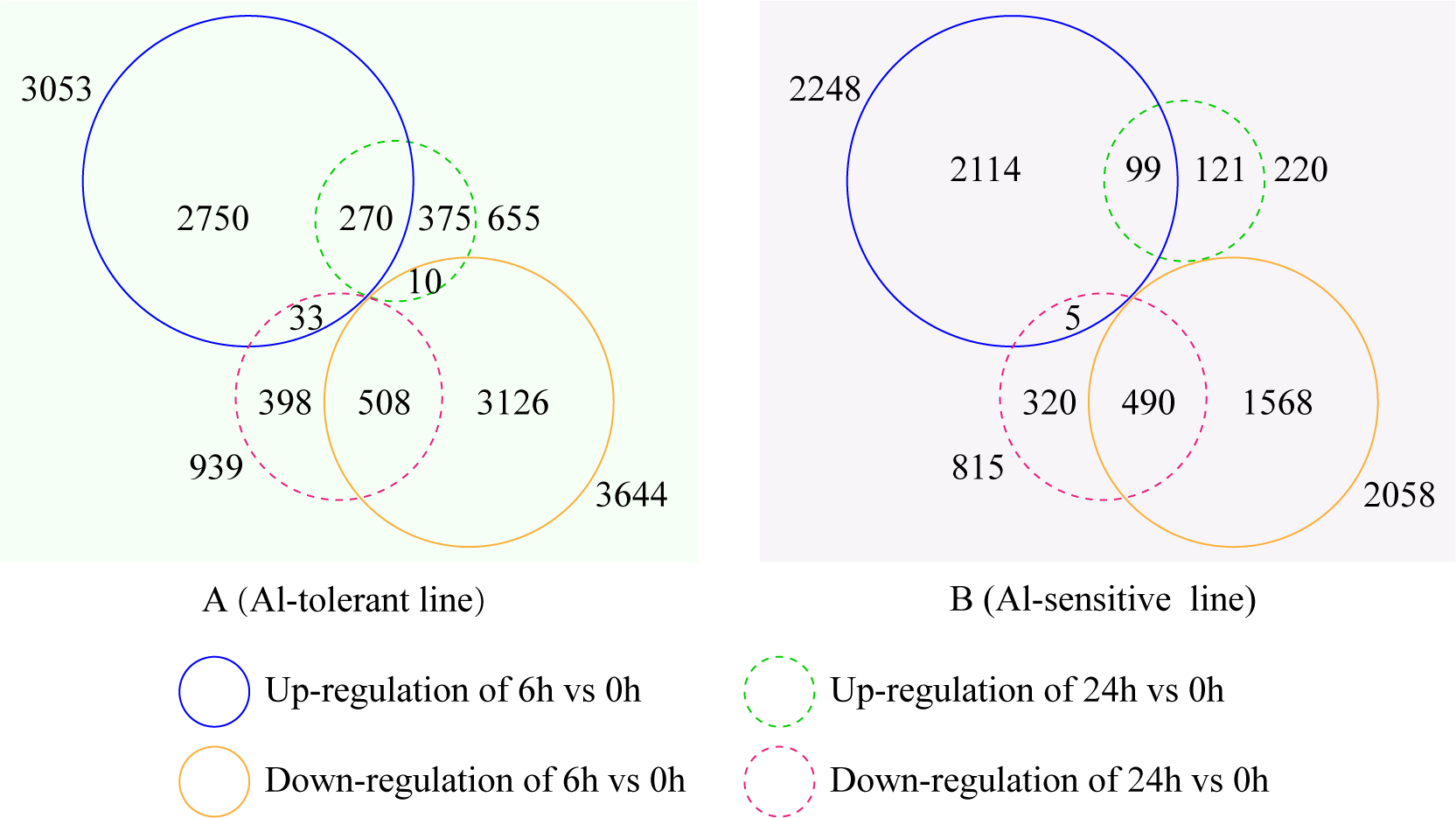

Supplement: Supplementary file 2 — Additional file 2. [file 12870_2022_3508_MOESM2_ESM.tif]

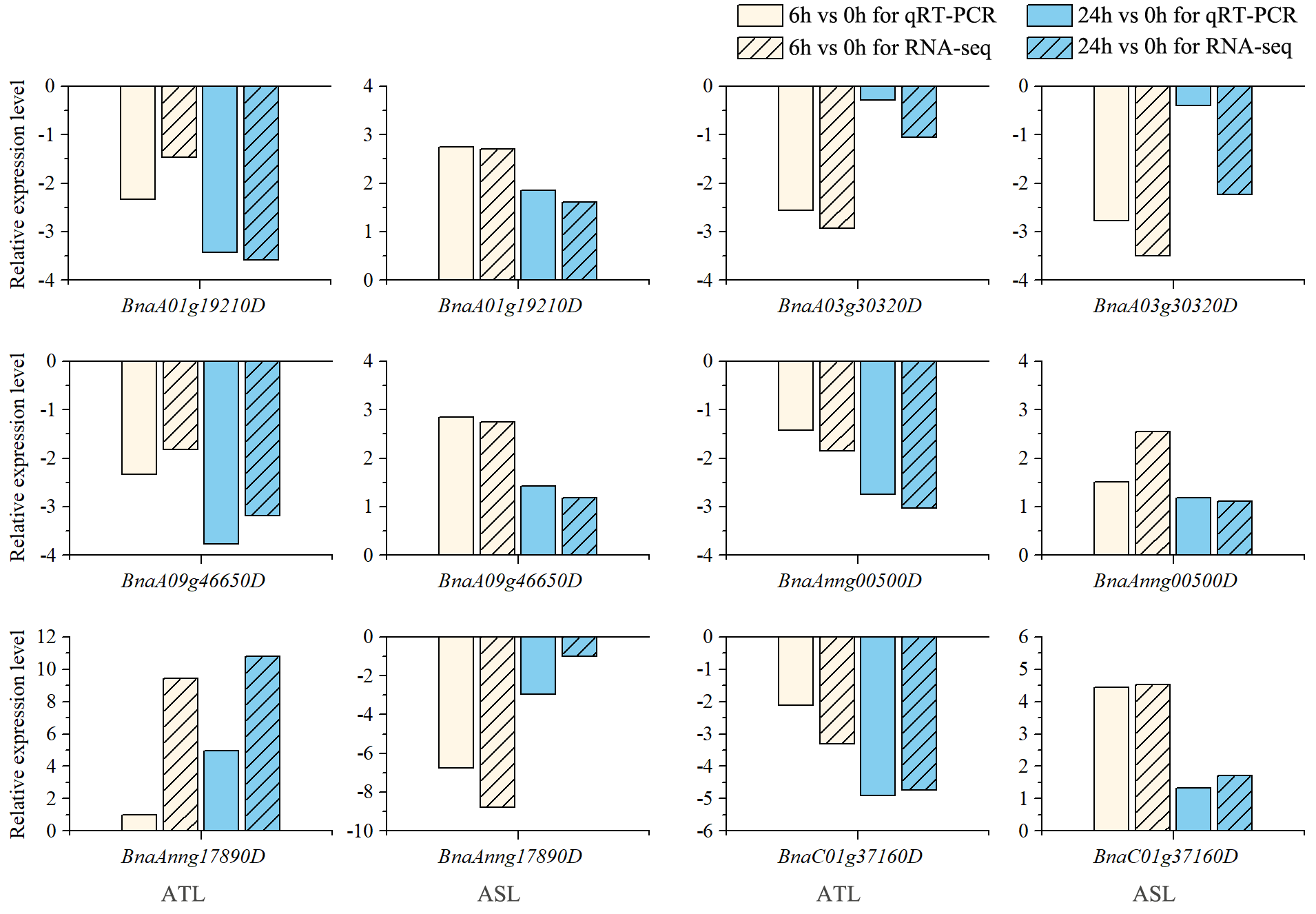

Supplement: Supplementary file 3 — Additional file 3. [file 12870_2022_3508_MOESM3_ESM.tif]

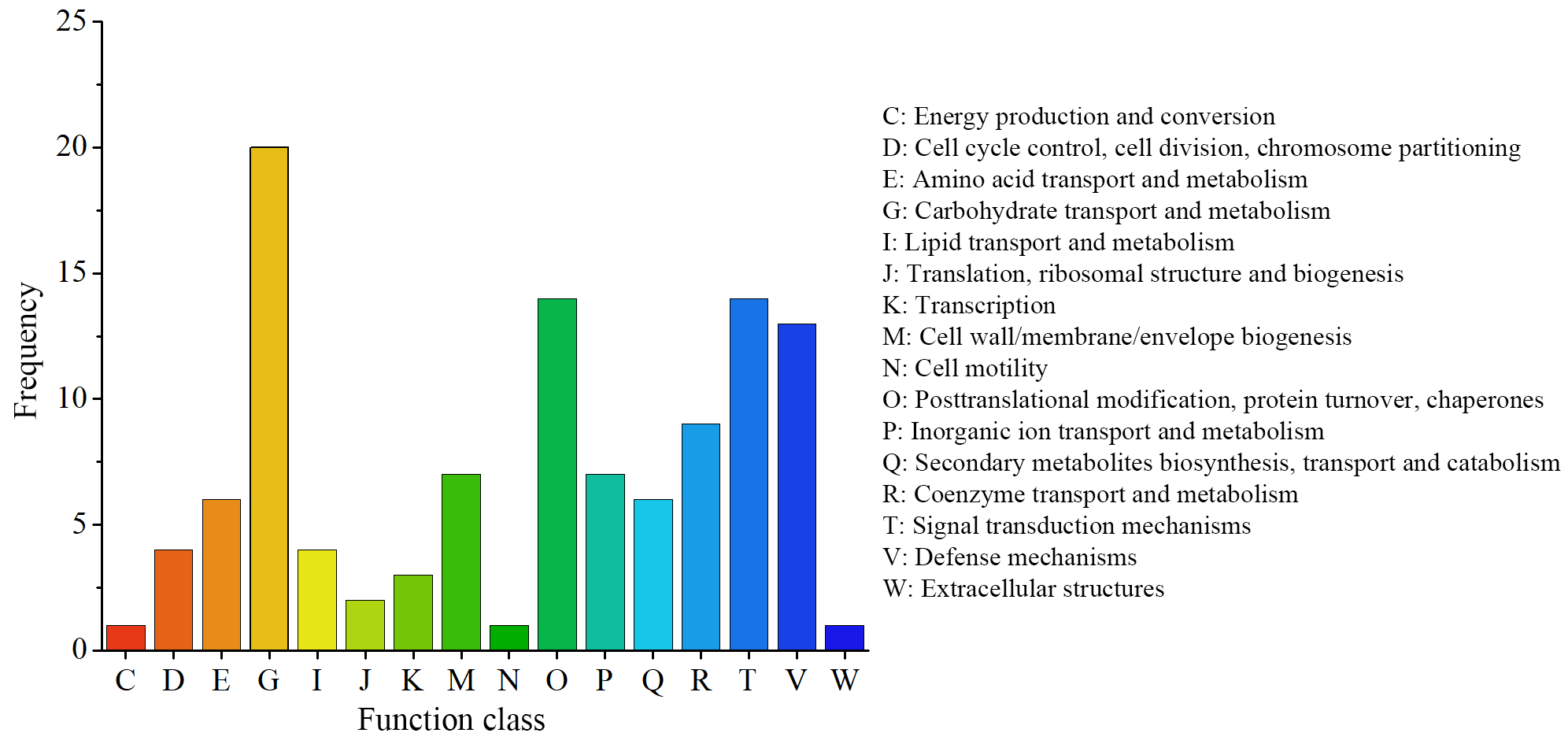

Supplement: Supplementary file 4 — Additional file 4. [file 12870_2022_3508_MOESM4_ESM.tif]

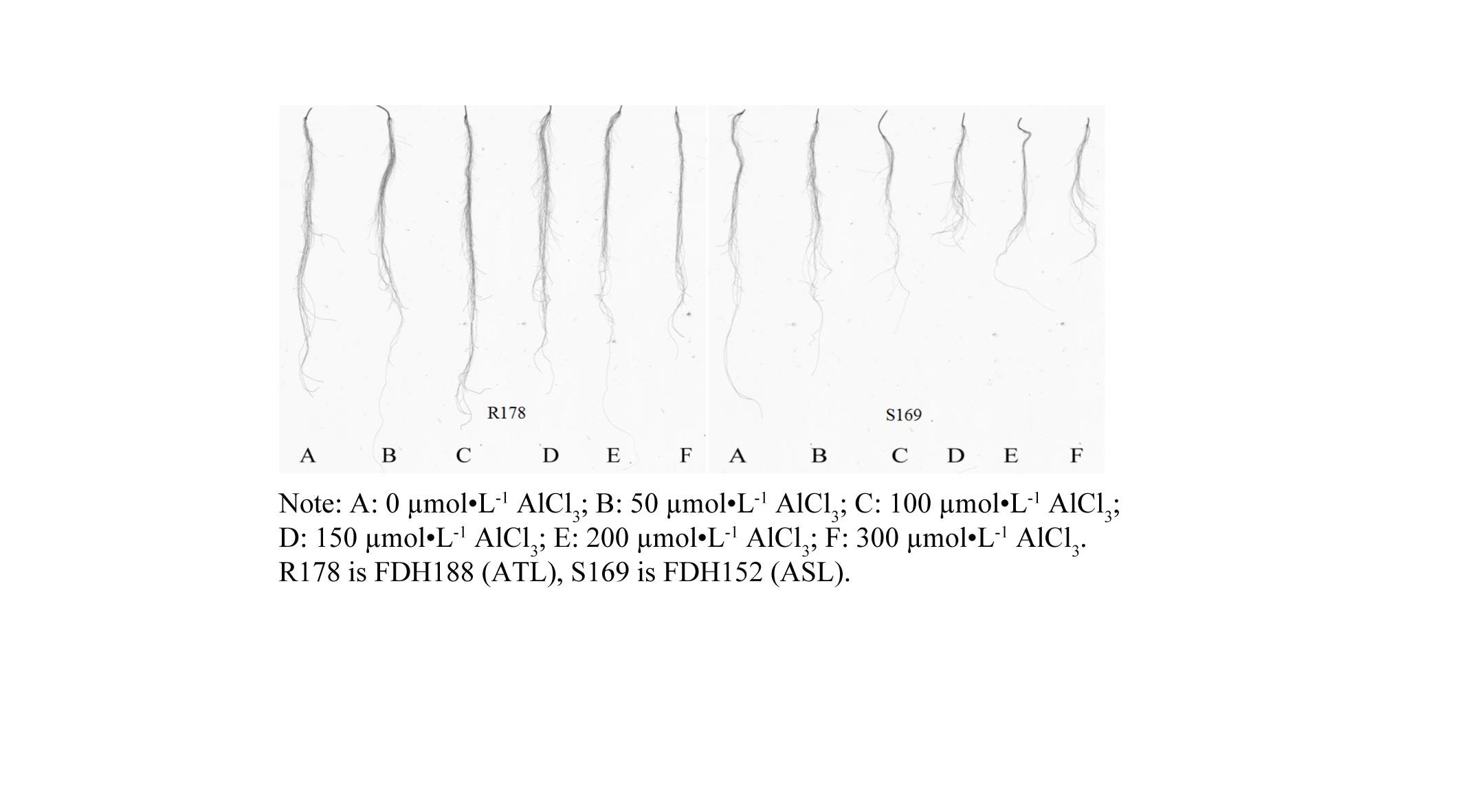

Supplement: Supplementary file 5 — Additional file 5. [file 12870_2022_3508_MOESM5_ESM.tif]
