# Supplementary material for: Integration of GWAS and transcriptome analyses to identify SNPs and candidate genes for aluminum tolerance in rapeseed (Brassica napus L.)
Source: BMC Plant Biol. 2022 Mar 21;22:130. doi: 10.1186/s12870-022-03508-w (PMC8935790; doi:10.1186/s12870-022-03508-w)
Supplement: Supplementary file 6 — Additional file 6. [file 12870_2022_3508_MOESM6_ESM.docx]

Table S1 – Statistical analysis of phenotypic traits under CK and T in *Brassica napus*

| Trait | Treatment | Mean | Min | Max | SD | CV/% |
| --- | --- | --- | --- | --- | --- | --- |
| Fresh weight above ground / g | CK | 0.384 | 0.064 | 3.681 | 0.401 | 104.2 |
|  | T | 0.378 | 0.029 | 3.599 | 0.445 | 117.8 |
| Root average diameter / | CK | 0.453 | 0.295 | 0.746 | 0.079 | 17.3 |
|  | T | 0.448 | 0.299 | 0.775 | 0.077 | 17.2 |
| Root fresh weight / g | CK | 0.039 | 0.007 | 0.403 | 0.042 | 105.3 |
|  | T | 0.044 | 0.005 | 0.386 | 0.046 | 103.3 |
| Root elongation / cm | CK | 3.066 | 0.075 | 9.975 | 1.675 | 54.6 |
|  | T | 3.274 | 0.333 | 9.975 | 1.650 | 50.4 |
| Total root surface area / cm^2^ | CK | 14.478 | 6.984 | 37.740 | 4.091 | 28.3 |
|  | T | 15.058 | 6.071 | 58.317 | 4.973 | 33.0 |
| Total root tip | CK | 500.616 | 184 | 1208 | 171.225 | 34.2 |
|  | T | 574.320 | 301 | 1197 | 170.488 | 29.7 |
| Total root volume / cm^3^ | CK | 0.167 | 0.047 | 0.421 | 0.062 | 36.8 |
|  | T | 0.173 | 0.049 | 0.972 | 0.082 | 47.5 |
| Total root length / cm | CK | 103.017 | 52.980 | 270.886 | 26.352 | 25.6 |
|  | T | 106.981 | 54.459 | 287.544 | 27.826 | 26.0 |
